# Predicting Arm Nonuse in Individuals with Good Arm Motor Function after Stroke Rehabilitation: A Machine Learning Study

**DOI:** 10.3390/ijerph20054123

**Published:** 2023-02-25

**Authors:** Yu-Wen Chen, Yi-Chun Li, Chien-Yu Huang, Chia-Jung Lin, Chia-Jui Tien, Wen-Shiang Chen, Chia-Ling Chen, Keh-Chung Lin

**Affiliations:** 1 School of Occupational Therapy, National Taiwan University College of Medicine, 17, F4, Xu-Zhou Road, Taipei 100, Taiwan; 2 Department of Speech Language Pathology and Audiology, National Taipei University of Nursing and Health Sciences, 365, Mingde Road, Taipei 112, Taiwan; 3 Department of Occupational Therapy, I-Shou University College of Medicine, 8, Yida Road, Jiaosu Village, Yanchao District, Kaohsiung 824, Taiwan; 4 Department of Physical Medicine and Rehabilitation, College of Medicine, National Taiwan University, Taipei 10048, Taiwan; 5 Department of Physical Medicine and Rehabilitation, National Taiwan University Hospital, Taipei 10048, Taiwan; 6 Institute of Biomedical Engineering and Nanomedicine, National Health Research Institutes, 35, Keyan Road, Zhunan District, Miaoli 350, Taiwan; 7 Department of Physical Medicine and Rehabilitation, Chang Gung Memorial Hospital at Linkou, 5 Fusing Street, Gueishan District, Taoyuan 333, Taiwan; 8 Graduate Institute of Early Intervention, College of Medicine, Chang Gung University, 259 Wenhua 1st Road, Gueishan District, Taoyuan 333, Taiwan; 9 Division of Occupational Therapy, Department of Physical Medicine and Rehabilitation, National Taiwan University Hospital, 7 Chung-Shan South Road, Taipei 100, Taiwan

**Keywords:** stroke rehabilitation, arm nonuse, predictors, chronic stroke, machine learning

## Abstract

Many stroke survivors demonstrate arm nonuse despite good arm motor function. This retrospective secondary analysis aims to identify predictors of arm nonusers with good arm motor function after stroke rehabilitation. A total of 78 participants were categorized into 2 groups using the Fugl-Meyer Assessment Upper Extremity Scale (FMA-UE) and the Motor Activity Log Amount of Use (MAL-AOU). Group 1 comprised participants with good motor function (FMA-UE ≥ 31) and low daily upper limb use (MAL-AOU ≤ 2.5), and group 2 comprised all other participants. Feature selection analysis was performed on 20 potential predictors to identify the 5 most important predictors for group membership. Predictive models were built with the five most important predictors using four algorithms. The most important predictors were preintervention scores on the FMA-UE, MAL–Quality of Movement, Wolf Motor Function Test-Quality, MAL-AOU, and Stroke Self-Efficacy Questionnaire. Predictive models classified the participants with accuracies ranging from 0.75 to 0.94 and areas under the receiver operating characteristic curve ranging from 0.77 to 0.97. The result indicates that measures of arm motor function, arm use in activities of daily living, and self-efficacy could predict postintervention arm nonuse despite good arm motor function in stroke. These assessments should be prioritized in the evaluation process to facilitate the design of individualized stroke rehabilitation programs to reduce arm nonuse.

## 1. Introduction

Stroke is a leading cause of disability [1] that can lead to chronic arm impairment [2,3]. Reduced arm function negatively affects stroke survivors’ quality of life [4]. A significant proportion of stroke survivors perceived nonuse in the affected arm as a major problem at 4 years after stroke [2]. Studies have reported constraint-induced movement therapy and its variants were effective in reducing arm nonuse and improving functional independence and quality of life in individuals with stroke [5,6]. Neurobiological changes corresponding to improvement in arm use after constraint-induced therapy were also observed with brain imaging [6]. Recently, researchers also advocated for a paradigm shift in stroke rehabilitation toward self-practice to reduce arm nonuse following motor rehabilitation [7]. However, early identification of individuals with stroke who may benefit the most from these programs is challenging. In the context of precision medicine, if individuals who are likely to develop arm nonuse can be identified early in the course of rehabilitation, the information would be able to guide and shape the individualized rehabilitation program.

Researchers have strived to investigate the prediction of arm nonuse in individuals with stroke. Many factors were found or proposed as being associated with the development of arm nonuse across the phases (i.e., acute, subacute, and chronic) after stroke. The factor with the most empirical support was upper-limb motor function. Substantial evidence supports the association between baseline or postintervention arm motor function and the amount of use or postintervention amount of use in the affected limb [8,9,10]. In the acute phase, age, stroke severity, sensory impairment, degree of disability at discharge, and hand grip strength were associated with the development of arm nonuse at 90 days after discharge [11,12]. In chronic stroke, upper-limb motor dysfunction, dependence in activities of daily living, and participants’ own perceived upper-limb function were associated with activities in the affected upper limb measured by wrist-worn accelerometers [13,14]. Self-efficacy was found to predict arm nonuse in a small group of participants [8] and to moderate the predicting relationship between upper-limb motor function and ratings of daily upper-limb use [15]. Emotional state, such as depression, was discussed as a potential factor in the context that it may affect compliance with constraint-induced movement therapy [16]. Other factors for daily upper-limb use after stroke rehabilitation, such as motivation, health behaviors, and environmental support, were also proposed but not explicitly tested [17].

Despite strong evidence suggesting the association between upper-limb motor function and daily use, good upper-limb motor function does not translate directly into more use of the affected limb. Studies have identified participants who demonstrated low daily use of the affected limb despite having good upper-limb motor function [18,19]. Some patients showed improvement in upper-limb function after rehabilitation, but continued to demonstrate low daily use of the affected limb [13,20]. This makes the prediction of nonuse a particularly challenging task. There are a few possible reasons behind the challenge. First, the relationship between arm function and arm use after stroke may be nonlinear [21,22] and may be subject to the moderating effects of other factors [15]. Second, the theoretical background that arm nonuse after stroke is learned [23] predicts that upper-limb motor function cannot be a sole predictor. Findings from earlier studies supported factors other than upper-limb motor function played a role in daily upper-limb activity [13,18]. Nevertheless, it may be a strong enough predictor to mask others, making it difficult to detect ancillary but important factors using traditional regression models.

The advances in artificial intelligence have provided us with another tool for data analysis, machine learning. Unlike traditional statistics, machine learning uses multidimensional linear and nonlinear methods to find patterns in the data [24], striving to achieve as high an accuracy as possible. It has the potential to identify factors that have nonlinear, complex relationships with upper-limb activity and achieve a high predicting capacity.

This study used machine learning methods to investigate predictors that can help identify individuals that were likely to develop arm nonuse but had good arm motor function after stroke rehabilitation. As discussed in the previous paragraphs, many potential predictors could be considered. We searched our database accumulated in the past few years to locate data with a set of likely predictors according to the literature. Furthermore, we grouped our participants in the database into two predefined groups according to their postintervention assessment results: those with low upper-limb use and good upper-limb motor function in the affected arm, and all others. We were interested in using preintervention measures to predict which patients would be likely to develop arm nonuse despite good upper-limb motor function after intervention. We hypothesize that measures other than motor function will emerge as important predictors for the classification. By doing so, we anticipate the results will inform clinical care planning when addressing personal and environmental factors in addition to motor function in the design of client-centered individualized rehabilitation programs.

## 2. Materials and Methods

This study was a retrospective secondary analysis of data collected for a study conducted by our research laboratory. The protocol and available results were published elsewhere [25,26]. The Institutional Review Boards of participating hospitals approved the study. All participants gave their written informed consent at the enrollment into the study.

### 2.1. Study Participants

Participants of the original study were recruited from outpatient clinics participating in our clinical trials and were screened by certified occupational therapists before being enrolled. The inclusion criteria were (1) at least 3 months after the onset of a first-ever unilateral cerebral stroke; (2) age between 18 and 80 years; (3) baseline Fugl-Meyer Assessment Upper-Extremity (FMA-UE) scale score of between 16 and 53; (4) a baseline spasticity score of ≤3 on the Modified Ashworth Scale; and (5) ability to follow the study instructions. The exclusion criteria were serious vision, orthopedic, or other neurologic disorders or medical conditions that could influence participation. The inclusion and exclusion criteria were set considering potential participants’ ability to follow the therapy program and potential confounding factors to the experimental design of the original study [25]. Participants were treated with robotic-primed mirror therapy or robotic-primed bilateral upper-limb training. They received the intervention approximately 90 minutes a session, 3 sessions a week, for 6 weeks. Treatment sessions and assessments were conducted by certified occupational therapists who were trained to conduct treatment programs for the original study. Assessments were completed by blinded therapists before and immediately after the therapy and at a 3-month follow-up. This retrospective study used data collected at preintervention and postintervention.

### 2.2. Outcome Measures and Potential Predictors

Participants were divided into two groups defined using postintervention scores on the FMA-UE and the Motor Activity Log–Amount of Use (MAL-AOU). Group 1 included participants with good upper-limb motor function (FMA-UE ≥ 31) [27] and low daily upper-limb use (MAL-AOU ≤ 2.5) [28]. All other participants were placed in group 2, including those with good upper-limb motor function and good daily upper-limb use, and those with low upper-limb motor function and low daily upper-limb use. Group label was the predicted variable for the machine learning models.

There were three categories of potential predictors for the machine learning models: (1) participant demographics: age, sex, years of education, and handedness percentage measured by the Edinburgh Handedness Inventory; (2) clinical characteristics: side of hemiplegia, time since stroke, diagnosis (hemorrhagic or ischemic), and the National Institutes of Health Stroke Scale score; and (3) baseline assessment scores: FMA-UE, Box and Block Test, the revised Nottingham Sensory Assessment–Tactile Sensation, the revised Nottingham Sensory Assessment–Proprioception, Wolf Motor Function Test-Time (WMFT-Time), WMFT-Quality (WMFT-Quality), MAL-AOU, MAL–Quality of Movement (MAL-QOM), Stroke Impact Scale-Hand, Stroke Impact Scale-Emotion, Functional Independence Measure (FIM), and Stroke Self-Efficacy Questionnaire (SSEQ). The baseline assessment scores were selected to reflect all three domains of the International Classification of Functioning, Disability and Health and to include factors with a suggested association with arm nonuse after stroke. The assessment tools are briefly described below.

The FMA-UE [29] is a measure of movement, reflex, and coordination of the upper limb. It contains 33 items rated by the clinician. The total score ranges from 0 to 66, with the higher scores indicating better motor function.

The Box and Block Test [30] is a test of manual dexterity. In the test, participants move blocks from one compartment to the other in a box with the affected hand in one minute. The score is number of blocks successfully transferred.

The revised Nottingham Sensory Assessment [31] is a short version of the Nottingham Sensory Assessment [32]. The assessment tests the sensory modalities of tactile sensation, proprioception, and stereognosis.

The WMFT [33] measures the upper-limb motor ability to perform structured tasks. The time needed to complete a given task (WMFT-Time) and the quality of upper-limb movement during the task (WMFT-Quality) are rated by the clinician on a scale of 0 (unable to complete) to 5 (no difficulty at all).

The MAL [34] is a patient-reported outcome measure designed to assess daily use of the affected upper limb outside the clinic in individuals with stroke. The 30 items of daily activities are rated on a scale of 0 (not used) to 5 (same as pre-stroke/normal). Participants rate the amount that the affected limb is used in each task (MAL-AOU), and the quality of movement of the affected limb in performing the task.

The Stroke Impact Scale [35] is a patient-reported global measure of activity and participation in individuals recovering from stroke. The SIS has eight domains: strength, hand function, mobility, activities of daily living, emotion, memory, communication, and social participation. The items are rated on the scale of 1 to 5, with a score of 5 indicating no difficulty completing the task. The summation score for each domain is transformed to a 100-point scale.

The FIM [36] is a widely used assessment for activities of daily living with 18 items that assess the areas of self-care, continence, mobility, transfer, communication, and cognition. The items are rated on a scale of 1 (total assistance) to 7 (complete independence).

The SSEQ [37] is a measure of an individual’s self-perceived level of confidence in performing functional tasks. Its 13 items are rated on the scale of 0 (not at all confident) to 10 (very confident).

### 2.3. Data Analysis

**Figure 1:**
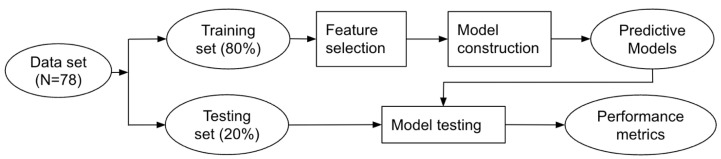
Diagram of the flow chart for data analysis.

Data analysis was performed using the Python Programing Language (RRID: SCR_008394) with the packages Sklearn 1.0 (RRID:SCR_019053) and Imblearn 0.8 (RRID: SCR_021698). Variable normality check was performed with the Shapiro–Wilk test. Figure 1 presents a diagram of our data analysis with machine learning. The data set was first split into two sets. The training set contained 80% of data and was used to develop predictive models, including feature selection, data preprocessing, and training and tuning of the models. The testing set of 20% was never seen by the models until performance testing to ensure that the testing data did not influence any model decision.

To identify the best predictors, we performed a feature selection analysis using mutual information [38]. The five predictors with the highest mutual-information gain were selected to build predictive models with four machine learning algorithms: classification and regression tree, k-nearest neighbors, logistic regression, and support vector machine. These algorithms are widely used in the classification tasks for health care data and are relatively simpler models, which we postulated were appropriate for our small sample size. They have also yielded good performance in studies on prediction models for stroke rehabilitation outcome [39,40,41,42,43,44].

The classification and regression tree is a decision-tree classifier that starts at the tree root and iterates splitting of the data on the feature that results in the largest information gain. The iteration stops when the tree leaves are pure or when experimenter pre-specified criteria have been met. The k-nearest neighbors is a distance-based method, where a data point is classified based on a majority vote of its k-nearest neighbors, where k is a number. The k-nearest neighbors is a special type of method in that the model does not “train” on the training data, but rather memorizes the training data, and looks for the majority vote in the training data to classify a testing data point. Logistic regression is a classification method that uses a sigmoid function to calculate the probability that a data point belongs to a class. It tends to perform well with linearly separable classes and allows the experimenter to set a regularization term to prevent it from overfitting. The support vector machine is a powerful and widely used learning algorithm. It learns by projecting data to higher dimensions and maximizing the distance between the separating hyperplane and the closest training data point [45].

During model construction, the synthetic minority oversampling technique was used to avoid the impact of imbalanced classes [46]. In machine learning, the algorithm strives to achieve high prediction accuracy and could over-focus on finding patterns in the majority class. This could result in a high overall prediction accuracy at the expense of low accuracy in the minority class. In the synthetic minority oversampling technique procedure, the minority class is oversampled by creating synthetic examples along the line segments joining any or all of the five nearest neighbors. The synthetic minority oversampling technique has been found to improve prediction performance in data with imbalanced classes [46]. Values of the predictors were also standardized to prevent the models from favoring predictors on scales of larger numbers [45]. 

Models were tuned with the grid search procedure with stratified 10-fold cross-validation to identify optimal hyperparameters based on classification accuracy. For classification and regression tree, maximum depth was tuned, and the tuning procedure determined 10 as the best value. For k-nearest neighbors, the number of neighbors and distance weight were tuned, and the procedure determined 3 neighbors and “uniform” as the best values. For logistic regression, the maximum number of iterators and the value of C were tuned, and 0.001 and 100 were the best values. For support vector machine, the kernel and the value of C were tuned, and the procedure determined the radial basis function and 10 to be the best values. Other hyperparameters were set to their defaults.

After the models were constructed, the testing set was used to test model performance. Performance was evaluated using a standard set of metrics: classification accuracy, the area under the receiver operating characteristic curve (AUC), specificity, sensitivity, negative predictive value, and positive predictive value.

## 3. Results

### 3.1. Participant Demographics and Assessment Results

We located 82 potential participants from the database. Four had missing data and were excluded from the analysis. As a result, 78 participants were included in this study. Table 1 presents participant demographics and preintervention clinical assessment scores. Shapiro–Wilk tests found that Age (W = 0.99, *p* = 0.51), SIS-Emotion (W = 0.97, *p* = 0.09), and WMFT-Quality (W = 0.98, *p* = 0.34) were normally distributed. Means and standard deviations were presented for these three variables. All other continuous variables had non-normal distributions (*p* ≤ 0.01).

**Figure 2:**
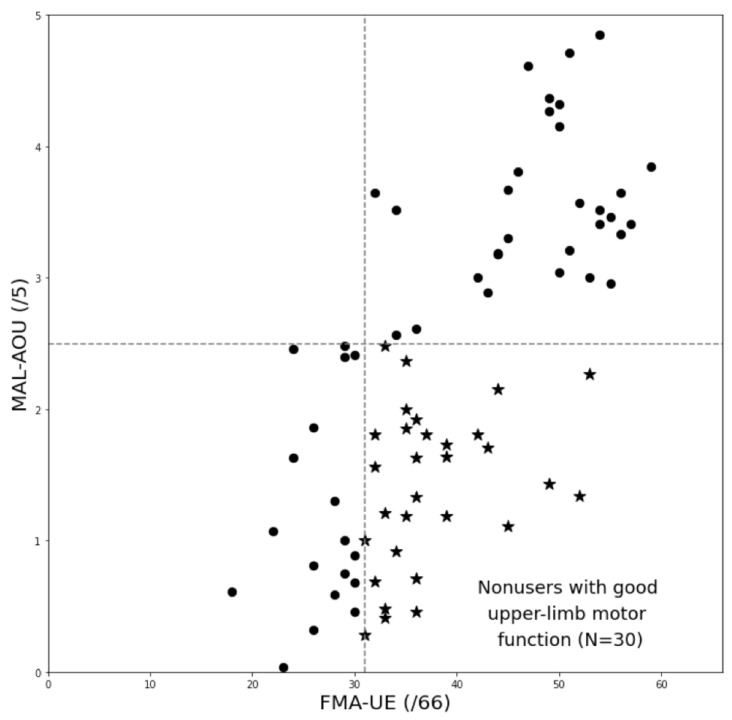
Scatterplot of the grouping schema. The stars (★) in the graph represent participants in Group 1 (nonuse with good arm motor function), and the dots (●) represent those in Group 2 (all others).

There were 30 participants (nonusers with good arm motor function) in group 1 and 48 participants (participants with matched arm motor function and daily use) in group 2. Figure 2 presents the two groups on the scatterplot with FMA-UE on the horizontal axis and MAL-AOU on the vertical axis. The two measures were correlated (Spearman’s rho = 0.69, *p* < 0.001).

### 3.2. Most Important Predictors

Table 2 presents the results for the feature selection analysis. The predictors were sorted in the order of their mutual information gain, and potential predictors with a gain of zero are listed together in the last row. The most important predictors for the group membership were baseline FMA-UE (gain = 0.20), followed by three measures of baseline upper-limb activity, MAL-QOM (gain = 0.12), WMFT-Quality (gain = 0.10), and MAL-AOU (gain = 0.08), and baseline SSEQ (gain = 0.05).

### 3.3. Predictive Models

Table 3 reports performance metrics of the predictive models built with the four algorithms and the five selected predictors identified by the feature selection procedure. The model with the best prediction performance was the k-nearest neighbors model, which had a prediction accuracy of 0.94 and AUC of 0.97. The second-best model was the support vector machine model, with a prediction accuracy of 0.88 and AUC of 0.95. Models built with classification and regression tree and logistic regression also yielded good classification performance.

## 4. Discussion

Despite the relationship between arm motor function and arm use after a stroke, many individuals continue to demonstrate low use of the affected arm even when their observed arm motor function has recovered to a certain level after rehabilitation [13,14,18,19,20]. In this study, we identified a group of participants who demonstrated arm nonuse after stroke rehabilitation, but had good arm motor function, and investigated preintervention predictors that could discriminate this group from other participants. We used machine learning to conduct the analysis appreciating its ability to find complex patterns in the data. We hypothesized that factors other than preintervention motor function would be associated with arm nonuse, and the findings supported this hypothesis. In addition to FMA-UE, our feature selection analysis identified MAL-AOU, MAL-QOM, WMFT-Quality, and SSEQ as important predictors. Machine learning predictive models built with these five predictors could classify the participants as accurately as 94%.

Although we grouped our participants so that one of the groups contained participants with both high and low postintervention FMA-UE and MAL-AOU, the preintervention scores of these two measures were still important predictors for group membership. It is possible that the use of machine learning, which could find complex, nonlinear patterns in the data, enabled the classification. In this context, the results aligned with previous findings that arm nonuse or daily use of the affected arm after stroke rehabilitation could be predicted by baseline upper-limb motor function [10,15]. In addition, two recent cross-sectional studies also reported positive relationships between postintervention severity of arm impairment and extent of arm nonuse [8,9]. Both studies quantified arm nonuse using objective, clinical observations, while we followed the more widely adopted convention to define arm nonuse using the MAL, which is a patient-reported outcome measure. Despite methodological discrepancies among the cumulative studies, collective evidence suggests that baseline and postintervention arm motor function could be predictive of postintervention arm nonuse in stroke.

Notably, two measures of quality of upper-limb movement, MAL-QOM and WMFT-Quality, were determined as important predictors. MAL-QOM is a subjective, patient-reported outcome measure, whereas WMFT-Quality is a clinician-rated scale. The finding that both emerged as important predictors substantiates the predicting ability of preintervention quality of movement of the affected arm for postintervention arm use. To our knowledge, this is the first study to report relationships of quality of movement to arm nonuse in stroke survivors. The descriptive statistics may provide insight into the interpretation of the results. In our study sample, the medians and first and third quartiles of MAL-QOM were 0.53 and 0.32–0.82 for group 1 and 1.17 and 0.32–1.96 for group 2. A Mann–Whitney U test revealed a significant group difference (*p* = 0.009). Participants who had rated their quality of movement as lower at preintervention tended to have lower arm use at postintervention despite their good arm motor function. However, this group difference was not found in WMFT-Quality (*p* = 0.15 with the independent *t*-test), possibly due to a more complex relationship of this clinician-rated measure to group membership. Further research is needed to elucidate the interactions between the two measures and their additive or individual predicting ability for arm nonuse.

The finding that preintervention SSEQ was one of the most important predictors supports its value to predict arm functional use after stroke [8,15]. Ma et al. [15] reported that scores on the SSEQ moderated the predictive relationship between preintervention FMA-UE and postintervention MAL-AOU. Buxbaum and colleagues [8] reported self-efficacy as a predictor for arm nonuse in a small group of participants with chronic stroke. Greater self-efficacy was associated with reduced arm nonuse. Our study extended to use a larger sample and used machine learning in the attempt to understand the complex relationship between motor function and daily use of the affected arm. In our sample, the medians and first and third quartiles of preintervention SSEQ were 90.5 and 77.75–102.5 for group 1 and 105.0 and 92.75–116.25 for group 2. A Mann–Whitney U test revealed a significant group difference (*p* = 0.006). Thus, it appears that participants who demonstrated arm nonuse and good arm motor function at postintervention (group 1) had rated their self-efficacy as lower at preintervention. 

A previous study identified patient independence in activities of daily living as a predictor for arm use in chronic stroke [13]. This relationship was supported by our finding that the FIM was the sixth most important predictor, indicating potential roles of preintervention levels of independence in activities of daily living in the prediction of postintervention arm nonuse. Interestingly, however, when we added this predictor to our predictive models, none of the algorithms could yield better prediction accuracy than the models built with five predictors, indicating that this variable did not add information for the classification task. In the study of Bailey et al. [13], independence in activities of daily living was measured cross-sectionally at the same time as arm use, whereas we used preintervention levels of functional independence to predict postintervention arm use. The differential relationships between baseline and postintervention functional independence and postintervention arm nonuse should be studied further in future research.

In our feature selection procedure, we included potential predictors that were commonly used in prediction models for upper-limb activity and those that were proposed to affect its functional use, such as demographic and stroke characteristics, emotional status, and sensory impairment. However, the influence of these candidates was outweighed by motor function, use and quality of movement of the affected limb, and self-efficacy. Our findings and those of previous studies suggest that some of these commonly used or proposed factors are important predictors for arm nonuse in the acute stage [11,12] but not as important in the chronic stage.

The identification of predictors other than preintervention upper-limb motor function contributes to clinical application in stroke rehabilitation. Although previous studies have repeatedly identified poor arm motor function as an important predictor, arm nonuse has been observed in many patients with good motor function, indicating roles of other predictors. Our findings indicate that, in addition to upper-limb motor function, affected-arm use and quality of movement and self-efficacy could be used to accurately predict postintervention arm nonuse in chronic stroke. Accurate prediction of postintervention arm nonuse could guide rehabilitation planning and clinical decision making; clinicians could adopt certain treatments or strategies to prevent the development of arm nonuse [6,7,47]. Preintervention assessments could include tests of these aspects, to identify patients with a risk for arm nonuse. Regarding candidate tests, quality of movement in the affected limb could be assessed using a patient-reported outcome measure or a clinician-rated measure, as both were identified as important predictors in our study. 

Furthermore, machine learning methods could be used to construct predictive models when the relationships of potential predictors and arm use are complex. In a clinic, this could be performed by using a readily available, open-source tool such as Python (RRID: SCR_008394), R (RRID: SCR_001905), or the Waikato Environment for Knowledge Analysis (RRID: SCR_001214). Clinic-specific predictive models could be built and evaluated using data from individual clinics, and new data could be entered into the resulting models to predict the individual’s risk of developing arm nonuse. If an individual was identified as being likely to develop arm nonuse despite good motor function, rehabilitation programs could be tailored to address relevant factors and reduce arm nonuse.

This study has several limitations. First, the limited sample size posed risks to the machine learning analysis; the models were more susceptible to characteristics in the data such as class imbalance and the scales of the measures. In this context, we made efforts to minimize potential additive effects from the characteristics of the data. Second, although factors such as age and side of hemiplegia did not yield as high mutual information scores, they were potentially important predictors in samples drawn from other populations. For example, most of our participants were within the age range of upper 50s to 60s, and therefore the results should be interpreted with caution when applied to older patients. Furthermore, the trade-off of using machine learning methods is the lack of interpretability of the predictive models because of the black-box analysis. We provided supplement statistics in the Section 4. However, the accurate prediction of arm nonuse in individual clinics will be the most reliable by models trained using their own data. Lastly, as a secondary analysis, some potentially important predictors (e.g., neglect and real-life situations of the participants) were not included for study. Another potentially important factor was treatment methods. While this study focused on the potential predictors of baseline characteristics, treatment program allocation could have influenced arm daily use. Further research including additional factors and based on a larger sample is needed.

## 5. Conclusions

This study used machine learning to investigate the complex relationship between potential predictors and arm nonuse in chronic stroke after rehabilitation. The results identified a set of preintervention predictors that in combination could accurately identify participants who demonstrated arm nonuse in spite of their good arm function after rehabilitation. Assessments of arm motor function, arm use in activities of daily living, and self-efficacy should be prioritized in the evaluation process prior to the start of rehabilitation program, to identify individuals who are likely to develop arm nonuse despite good arm motor function after rehabilitation. The assessment result could inform the design of client-centered rehabilitation programs. Furthermore, machine learning may be a relevant tool for the construction of data-driven and clinic-specific predictive models for stroke rehabilitation outcome, especially when the predicting relationship is complex.

## Figures and Tables

**Table 1:** Participant demographics and preintervention assessment scores.

Participant Characteristics	Mean ± SD	Median (Q1; Q3)	n (%)
Demographics			
Age (years)	56.43 ± 11.05		
Male sex			53 (68)
Years of education		12.00 (9.00; 15.00)	
Edinburgh Handedness Inventory		100.00 (90.00; 100.00)	
Clinical Characteristics			
Right-sided hemiplegia			47 (60)
Time since stroke (months)		11.75 (6.25; 23.00)	
Hemorrhagic stroke diagnosis			35 (45)
NIHSS score		5.00 (3.00; 6.00)	
Baseline Assessment Scores			
FMA-UE		32.00 (26.00; 41.00)	
BBT		2.00 (0.00; 17.75)	
rNSA–Tactile		97.00 (59.50; 104.00)	
rNSA–Proprioception		19.00 (14.00; 21.00)	
WMFT-Time		10.80 (7.10; 15.17)	
WMFT-Quality	2.48 ± 0.57		
MAL-AOU		0.89 (0.45; 1.66)	
MAL-QOM		0.79 (0.32; 1.42)	
SIS-Hand		37.50 (15.00; 58.75)	
SIS-Emotion	64.53 ± 21.37		
FIM		109.00 (102.25; 113)	
SSEQ		101.00 (83; 110.75)	

Abbreviations: BBT, Box and Block Test; FIM, Functional Independence Measure; FMA-UE, Fugl-Meyer Assessment Upper Extremity Scale; MAL-AOU, Motor Activity Log–Amount of Use; MAL-QOM, Motor Activity Log–Quality of Movement; NIHSS, National Institutes of Health Stroke Scale; Q1, first quartile; Q3, third quartile; rNSA, Revised Nottingham Sensory Assessment; SIS, Stroke Impact Scale; SSEQ, Stroke Self-Efficacy Questionnaire; WMFT, Wolf Motor Function Test.

**Table 2:** Mutual information gain of the predictors, sorted by gain.

Predictor	MI Gain
Baseline FMA-UE	0.20
Baseline MAL-QOM	0.12
Baseline WMFT-Quality	0.10
Baseline MAL-AOU	0.07
Baseline SSEQ	0.05
Baseline FIM	0.03
Baseline BBT	0.02
Age	0.02
Side of hemiplegia	0.02
Years of education	0.01
Baseline SIS-Hand	0.003
Sex, Edinburgh Handedness Inventory, Diagnosis, NIHSS, Time since stroke, Baseline rNSA-proprioception, Baseline rNSA-Tactile sensation, Baseline WMFT-Time, Baseline SIS-Emotion	0

Abbreviations: BBT, Box and Block Test; FIM, Functional Independence Measure; FMA-UE, Fugl-Meyer Assessment Upper Extremity Scale; MAL-AOU, Motor Activity Log–Amount of Use; MAL-QOM, Motor Activity Log–Quality of Movement; MI, Mutual Information; NIHSS, National Institutes of Health Stroke Scale; rNSA, Revised Nottingham Sensory Assessment; SIS, Stroke Impact Scale; SSEQ, Stroke Self-Efficacy Questionnaire; WMFT, Wolf Motor Function Test.

**Table 3:** Model performance metrics.

Model	Accuracy	AUC	Specificity	Sensitivity	NPV	PPV
CART	0.88	0.83	1.00	0.67	0.83	1.00
KNN	0.94	0.97	1.00	0.83	0.91	1.00
LR	0.75	0.77	0.70	0.83	0.88	0.63
SVM	0.88	0.95	1.00	0.67	0.83	1.00

Abbreviations: AUC, area under the receiver operating characteristic curve; CART, classification and regression tree; KNN, k-nearest neighbors; LR, logistic regression; NPV, negative predictive Value; PPV, positive predictive value; SVM, support vector machine.

## Data Availability

The data presented in this study are available upon request from the corresponding author. The data are not publicly available because, according to the Personal Information Protection Act enacted in Taiwan, individualized data cannot be released for the protection of privacy.
